# Factors Shaping Medical Students’ Intentions Toward Neurosurgery: A Cross-Sectional Study

**DOI:** 10.7759/cureus.51107

**Published:** 2023-12-26

**Authors:** Sami F Almalki, Abdulelah S Almousa, Abdullah K Alarfaj, Muhannad M Alwadany, Abdullah F Al Wadani, Mohammed Yasser Ibrahim Daoud, Ossama M Zakaria

**Affiliations:** 1 Department of Neurosurgery, College of Medicine, King Faisal University, Al-Ahsa, SAU; 2 Department of Surgery, College of Medicine, King Faisal University, Al-Ahsa, SAU

**Keywords:** specialty selection, influential factors, medical students, neurosurgery, medical education

## Abstract

Background

Choosing a medical specialty poses a significant challenge for students, with initial fascinations often evolving during their academic journey. Despite its inherent appeal, neurosurgery faces hesitancy among undergraduate students, potentially due to perceived difficulties and time demands. This study aims to investigate the factors influencing medical students’ intentions toward neurosurgery at two institutions in the eastern province of Saudi Arabia.

Methodology

A cross-sectional study design was employed, utilizing a validated, anonymous questionnaire distributed electronically to medical students and interns in the eastern province of Saudi Arabia. The questionnaire comprised two sections, namely, demographic and academic profiles, and participants’ intentions, knowledge, and attitudes toward neurosurgery. Data analysis involved descriptive statistics, and chi-square tests to explore relationships and identify significant predictors.

Results

Of the participants, 197 (34.1%) expressed interest in neurosurgery, but only 94 (16.3%) had a comprehensive understanding of the field. Motivations included high income, specialty prestige, and positive impact on patients, while stress and work-life balance were common deterring factors. Age and academic years were associated with a gradual decrease in interest, except for initial medical school students. Participants were attracted to innovative technological aspects, while high competition and neurophobia were deterring factors.

Conclusions

This study provides a comprehensive analysis of determinants influencing medical students’ and interns’ interest in neurosurgery. Early exposure, clinical training, and personal motivations play significant roles in shaping career preferences. Challenges, such as perceived difficulties and concerns related to work-life balance, need targeted interventions to enhance neurosurgery’s attractiveness. Considerations extend beyond technical and academic facets to encompass personal and lifestyle dimensions.

## Introduction

Choosing a career path in medical specialties represents a significant challenge and concern for medical students, as their initial fascinations often evolve during their academic journey. Clinical training plays a pivotal role in shaping students’ perceptions and career intentions, as they gain deeper insights into various medical specialties [1-3]. Despite the inherent appeal and dynamism of neurosurgical specialties, hesitancy among undergraduate students to pursue this field has been observed [4,5]. This hesitation may stem from perceived difficulties and time and effort demands, making neurosurgery seem less applicable compared to other disciplines [6].

The decision to specialize in neurosurgery is influenced by a complex interplay of factors, including undergraduate studies, clinical exposure to patients, personal ambition, character, the duration of postgraduate studies, gender, and expected income [7,8]. Recent reports highlight neurosurgery as a highly sought-after and competitive specialty for residency programs, emphasizing its significance in the medical landscape [8].

In the local context, neurosurgical training entails a rigorous process, encompassing six years of residency training that focuses on the theoretical foundations of neurosciences, honing surgical skills, and mastering both basic and advanced neurosurgical procedures. Graduates from this program are equipped to practice as general neurosurgeons. Subspecialization in neurosurgery requires an additional two to three years, culminating in a total commitment of eight to nine years for those aspiring to become highly specialized in the field. The primary objective of this study is to shed light on medical students’ intentions to choose neurosurgery as a professional career.

## Materials and methods

Study design

This study utilized an exploratory cross-sectional design, employing an anonymous survey questionnaire to explore the factors influencing medical students’ and interns’ decisions regarding pursuing a career in neurosurgery. Participants were electronically invited to complete the questionnaire, with a strong emphasis on ensuring anonymity and fostering open and honest responses. The survey was disseminated via email to medical students across various academic levels and interns. The study commenced on May 1, 2023, and remained accessible for 14 days.

Study participants

The participants in this study comprised medical students at various academic levels and interns from both King Faisal University and Imam Abdulrahman Bin Faisal University in the eastern province of Saudi Arabia. The participants encompassed a broad spectrum of academic levels, with no specific exclusion criteria.

Questionnaire development

The questionnaire was structured into two sections. The first section focused on participants’ demographic and academic profiles, encompassing six questions related to age, gender, and additional inquiries about their academic backgrounds. The second section delved into participants’ intentions, knowledge, and attitudes toward neurosurgery, featuring 11 questions. The questionnaire was designed based on expert input and a literature review of previously published studies. In the development of our questionnaire, we implemented a comprehensive validation process to ensure the instrument’s reliability and relevance to the study’s objectives. Drawing upon the expertise of professionals in the field, including faculty members specializing in neurosurgery, medical education, and survey design, the questionnaire underwent expert reviews. These experts provided critical feedback, contributing to the refinement of the questionnaire in terms of clarity, relevance, and comprehensiveness. Additionally, to assess the instrument’s practical applicability and identify potential ambiguities, a pilot test was conducted with a small sample of medical students. The feedback obtained from the pilot test was instrumental in further refining the questionnaire to enhance its clarity and ensure that participants’ responses aligned accurately with the intended objectives of the study.

Statistical analysis

Statistical analysis of the collected data was conducted using SPSS version 26 (IBM Corp., Armonk, NY, USA). Descriptive statistics, including frequencies and percentages, were employed to present demographic and academic profile information. Normality tests, specifically the Shapiro-Wilk and Kolmogorov-Smirnov tests, were performed to assess the distribution of data. Given the categorical nature of the variables, the chi-square test was chosen to explore the relationship between having an interest in neurosurgery and sociodemographic characteristics, as well as to identify attractors and deterring factors. Statistical significance was determined at a p-value of less than 0.05.

Ethical considerations

This study adhered to ethical guidelines, including the acquisition of informed consent from all participants. Participant identities remained confidential, and data were anonymized during analysis. Ethical approval for this study was obtained from King Faisal University, ensuring compliance with ethical standards and participant protection. The Institutional Review Board of King Faisal University issued approval (approval number: 2022-DEC-423).

## Results

Participant characteristics

A total of 578 medical students and interns participated in the study, ranging in age from less than 20 to 26 years. The cohort comprised 327 (56.6%) males and 251 (43.4%) females, resulting in a male-to-female ratio of 1.3 to 1. Of the participants, 287 (49.7%) were affiliated with King Faisal University, while the remaining 291 (50.3%) were from Imam Abdulrahman Bin Faisal University. Additionally, 130 (22.5%) respondents were medical interns. Notably, 228 (39.4%) participants had a grade point average rating between 4.00 and 4.49 (Table 1).

**Table 1:** Basic demographic characteristics of the medical students (n = 578). The table displays the basic demographic characteristics of the study participants. Data are presented as frequency (n) and percentage (%). Percentages are calculated based on the total number of participants (N = 578).

Study data		Frequency (n)	Percentage (%)
Age group	<20 years	149	25.8%
	20–22 years	179	31.0%
	23–26 years	250	43.3%
Gender	Male	327	56.6%
	Female	251	43.4%
University	King Faisal University	287	49.7%
	Imam Abdulrahman Bin Faisal University	291	50.3%
Academic year	Second year	89	15.4%
	Third year	69	11.9%
	Fourth year	87	15.1%
	Fifth year	105	18.2%
	Sixth year	98	17.0%
	Internship	130	22.5%
Grade point average	3.0–3.49	3	0.50%
	3.5–3.99	71	12.3%
	4.0–4.49	228	39.4%
	4.5–4.74	216	37.4%
	4.75–5.00	60	10.4%

Interest in neurosurgery

In total, 197 (34.1%) participants expressed interest in pursuing neurosurgery as a future career. However, only 94 (16.3%) participants indicated that they had an understanding of neurosurgery as a career path. None of the participants had completed a degree or project in the field of neurosurgery (Table 2).

**Table 2:** Perception of medical students regarding neurosurgery as a future specialty (n = 578). The table presents the frequency and percentage of medical students’ perceptions regarding neurosurgery as a future specialty. Data are presented as frequency (n) and percentage (%). Percentages are calculated based on the total number of participants (N = 578).

Statement		Frequency (n)	Percentage (%)
Interested in a career in neurosurgery	No	381	65.9%
	Yes	197	34.1%
I understand what a career in neurosurgery involves	No	484	83.7%
	Yes	94	16.3%
I understand what is required of me to obtain a position in a neurosurgery residency program	No	557	96.4%
	Yes	21	3.6%
I have previously attended a neurosurgical conference/out-of-curriculum course	No	566	97.9%
	Yes	12	2.1%
I have completed an additional degree, student-selected component, or project related to neurosurgery	No	543	93.9%
	Yes	35	6.05%
I have received neurosurgical teaching during my medical education	No	310	53.6%
	Yes	268	46.4%

Motivations and deterring factors

Most participants expressed motivation for a neurosurgical career due to factors such as high income (n = 469, 81.1%), specialty prestige (n = 440, 76.1%), and the perceived positive impact on patients (n = 433, 74.9%). Conversely, stress (n = 550, 95.2%), lifestyle considerations, and work-life balance (n = 449, 77.7%) were commonly considered deterring factors (Table 3).

**Table 3:** Attractors and deterring factors to neurosurgical specialty (n = 578). The table presents the frequency and percentage of factors influencing medical students’ interest (attractors) or hesitancy (deterring factors) toward neurosurgery. Data are presented as frequency (n) and percentage (%). Percentages are calculated based on the total number of participants (N = 578).

Factors		Frequency (n)	Percentage (%)
Attractors	Income	469	81.1%
	The prestige associated with the specialty	440	76.1%
	Impact on patients “rewarding”	433	74.9%
	Variety of cases and types of patients	412	71.3%
	Academic field preference for this major	331	57.3%
	The geographic location of the training center	184	31.8%
	Research opportunities	157	27.2%
	I am interested in neuroscience	149	25.8%
	Technology and Innovation	129	22.3%
	Practical aspect, surgical techniques, or high skill required	111	19.2%
	The intensity of competition in the field	95	16.4%
	Successful placement of recent graduates into desired subspecialty fellowship	70	12.1%
	Number of shifts/on-calls	60	10.4%
	Having a role model with the same specialty	58	10.0%
Deterring factors	Stress	550	95.2%
	Lifestyle, work-life balance	449	77.7%
	The intensity of competition in the field	432	74.7%
	Long training time	298	51.6%
	Neurophobia	287	49.7%
	Practical aspect, long surgeries	255	44.1%
	Few training centers	242	41.9%
	Other career interests	221	38.2%
	For complex patients	220	38.1%
	Gender diversity	135	23.4%
	Risks	97	16.8%
	Limited job opportunities	71	12.3%
	Income	35	6.1%

Factors associated with interest in neurosurgery

The study explored factors associated with interest in neurosurgery, including age, academic years, and specific attractors and detractors. Increased age and academic years were associated with a gradual decrease in interest in neurosurgery as a career option. However, this trend was contradicted among initial medical school students, who exhibited a high level of interest in neurosurgery. Additionally, participants identified innovative technological aspects as highly attractive factors influencing the choice of neurosurgery, while factors such as high competition in the field and neurophobia were commonly cited as deterring factors (Table 4).

**Table 4:** Neurosurgery specialty interest by demographics, attractors, and deterring factors in medical students (n = 578). The table presents factors associated with interest in neurosurgery among study participants. Data are presented as frequencies (n) and percentages (%) for each category. The p-values, indicating statistical significance determined by the chi-square test, are provided, with the significance level set at p < 0.05.

Factor		Interest in neurosurgery		P-value
		Yes (N = 197)	No (N = 381)	
		n (%)	n (%)	
Age group	<20 years	96 (48.7%)	53 (13.9%)	<0.01
	20–22 years	60 (30.5%)	119 (31.2%)	
	23–26 years	41 (20.8%)	209 (54.9%)	
Gender	Male	108 (54.8%)	219 (57.5%)	0.54
	Female	89 (45.2%)	162 (42.5%)	
University	King Faisal University	110 (55.8%)	177 (46.5%)	0.03
	Imam Abdulrahman Bin Faisal University	87 (44.2%)	204 (53.5%)	
Academic year	Second to Fourth Year	127 (64.5%)	118 (31.0%)	<0.01
	Fifth and Sixth Year	54 (27.4%)	149 (39.1%)	
	Internship	16 (08.1%)	114 (29.9%)	
GPA	<4.0	32 (16.2%)	42 (11.0%)	0.140
	4.0–4.49	70 (35.5%)	158 (41.5%)	
	4.5–5.0	95 (48.2%)	181 (47.5%)	
Received neurosurgical teaching during medical education	No	136 (69.0%)	174 (45.7%)	<0.01
	Yes	61 (31.0%)	207 (54.3%)	
Attractors or deterring factors	Income	188 (95.4%)	281 (73.8%)	<0.01
	The prestige associated with the specialty	188 (95.4%)	252 (66.1%)	<0.01
	Impact on patients “rewarding”	145 (73.6%)	288 (75.6%)	0.60
	Variety of cases and types of patients	145 (73.6%)	267 (70.1%)	0.38
	Academic field preference for this major	105 (53.3%)	226 (59.3%)	0.17
	The geographic location of the training center	89 (45.2%)	95 (24.9%)	<0.01
	Research opportunities	74 (37.6%)	83 (21.8%)	<0.01
	I am interested in neuroscience	140 (71.1%)	09 (02.4%)	<0.01
	Technology and innovation	69 (35.0%)	60 (15.7%)	<0.01
	Practical aspect, surgical techniques, or high skill required	68 (34.5%)	43 (11.3%)	<0.01
	The intensity of competition in the field	82 (41.6%)	13 (03.4%)	<0.01
	Successful placement of recent graduates into desired subspecialty fellowship	23 (11.7%)	47 (12.3%)	0.82
	Number of shifts/On-calls	60 (30.5%)	0	<0.01
	Having a role model with the same specialty	28 (14.2%)	30 (07.9%)	0.02

## Discussion

The pursuit of a medical career is often a lifelong dream and primary motivation for many medical students. Neurosurgery, with its rapidly evolving innovative techniques, has become an enticing choice for aspiring young medical professionals. Technologies such as artificial intelligence and robotic surgery have significantly contributed to the prestige of neurosurgery, making it a captivating field for many medical students [9].

One prominent factor influencing the interest in neurosurgery is the dynamic increase in neurosurgeons’ income [10]. The specialty also plays a vital role in treating a significant number of cases, especially in low, middle, and resource-challenged settings. However, despite the demand, the number of practicing neurosurgeons in Saudi Arabia remains disproportionately low, emphasizing the need for continued growth and interest in this specialty [11]. To address the research intentions of students, particularly in middle-income countries with restrictive settings, the study by Begagic et al. becomes pertinent [12]. This research provides valuable insights into the current underutilization of students’ research potential in the field of biomedical sciences in Bosnia and Herzegovina. According to the findings, there is a significant gap in the students’ knowledge and participation in scientific research activities, reflecting a need for improvement [12]. These observations parallel our concerns about the scarcity of neurosurgeons and highlight a broader global issue regarding the underutilization of student research potential across various medical disciplines and geographical regions.

The study’s finding that 34.1% of participants expressed an interest in becoming neurosurgeons aligns with similar reports in the literature [13]. In our institute, early exposure to neurosciences and neurosurgical topics within the problem-based learning academic curriculum appears to significantly impact junior medical students’ interest in neurosurgery. However, a gradual decline in interest as academic grading advances suggests that external factors, such as perceived risks and malpractice concerns, may influence career choices. Exposure to malpractice concepts during forensic medicine courses, coupled with a broader understanding of the profession’s challenges, may contribute to a decline in interest among senior students [13-15].

Factors influencing interest and motivation in neurosurgery are multifaceted. While junior students may be more motivated by patients’ satisfaction, cure, and the allure of innovative technology, senior students and interns may weigh lifestyle difficulties, long training periods, and stress more heavily. Despite the challenges, students who perceive neurosurgery as a highly gratifying job that meets their ego and demands are more likely to find the specialty attractive [16].

The study identifies significant challenges and deterring factors that impact the choice of neurosurgery as a career. Factors such as the long journey, extensive training years, and stress are cited by a substantial percentage of participants as deterrents. This is consistent with findings from similar settings but underscores the importance of addressing these concerns to encourage interest and commitment to the field. Burnout emerges as a prevalent factor that detracts students from pursuing neurosurgery [17].

While the study provides valuable insights, it is essential to acknowledge its limitations. The cross-sectional design limits the ability to establish causal relationships, and the reliance on self-reported data introduces potential biases. Future research should explore the long-term impact of early exposure to neurosurgery, evaluate the effectiveness of interventions, and delve into the cultural and regional factors influencing career choices.

## Conclusions

This study provides a comprehensive analysis of the determinants influencing medical students’ and interns’ proclivity toward neurosurgery as a prospective career. Through an exploration of demographic, academic, and attitudinal dimensions, we elucidate the intricate factors that contribute to decision-making within this specialized domain. Our findings underscore the significance of early exposure, clinical training, and personal motivations in shaping career preferences. The identified challenges, including perceived difficulties and concerns related to work-life balance, underscore potential focal points for targeted interventions to enhance the attractiveness of neurosurgery. The considerations of prospective neurosurgeons extend beyond technical and academic facets to encompass personal and lifestyle dimensions.

## References

[1] Al-Zubi M, Ali MM, Alzoubi S (2021). Preference of and factors that influence future specialty among medical students in Jordan: a cross-sectional study. Ann Med Surg (Lond).

[2] Kaliyadan F, Amin TT, Qureshi H, Al Wadani F (2015). Specialty preferences of 1(st) year medical students in a Saudi medical school - factors affecting these choices and the influence of gender. Avicenna J Med.

[3] Khamees A, Awadi S, Al Sharie S, Faiyoumi BA, Alzu'bi E, Hailat L, Al-Keder B (2022). Factors affecting medical student's decision in choosing a future career specialty: a cross-sectional study. Ann Med Surg (Lond).

[4] Arnold MW, Patterson AF, Tang AS (2005). Has implementation of the 80-hour work week made a career in surgery more appealing to medical students?. Am J Surg.

[5] Woodrow SI, Gilmer-Hill H, Rutka JT (2006). The neurosurgical workforce in North America: a critical review of gender issues. Neurosurgery.

[6] Whitaker M (2018). The surgical personality: does it exist?. Ann R Coll Surg Engl.

[7] Patel V, Buchanan H, Hui M, Patel P, Gupta P, Kinder A, Thomas H (2018). How do specialist trainee doctors acquire skills to practice patient-centred care? A qualitative exploration. BMJ Open.

[8] van de Wiel MW, Van den Bossche P, Janssen S, Jossberger H (2011). Exploring deliberate practice in medicine: how do physicians learn in the workplace?. Adv Health Sci Educ Theory Pract.

[9] Heikkilä TJ, Hyppölä H, Vänskä J (2015). Factors important in the choice of a medical career: a Finnish national study. BMC Med Educ.

[10] Burford C, Hanrahan J, Ansaripour A (2019). Factors influencing medical student interest in a career in neurosurgery. World Neurosurg.

[11] Algahtani AY, Jamjoom AB, Al Rabie A, Jamjoom ZA (2021). Attrition and success rates in the Saudi Board of Neurosurgery: analysis of 115 consecutive residents who started training from 2001 to 2014. Cureus.

[12] Begagic E, Duzic N, Memic Z, Arandelovic N, Celebic A, Beculic H (2021). Usage of students' potential in biomedical and health care research in Bosnia and Herzegovina. Medeni Med J.

[13] Wilson MP, Pugh JA (2012). Increasing the appeal of neurosurgery to qualified medical students in Canada. Can J Neurol Sci.

[14] Agarwal N, Norrmén-Smith IO, Tomei KL, Prestigiacomo CJ, Gandhi CD (2013). Improving medical student recruitment into neurological surgery: a single institution's experience. World Neurosurg.

[15] Akhigbe T, Sattar M (2014). Attitudes and perceptions of medical students toward neurosurgery. World Neurosurg.

[16] Klimo P Jr, DeCuypere M, Ragel BT, McCartney S, Couldwell WT, Boop FA (2013). Career satisfaction and burnout among U.S. neurosurgeons: a feasibility and pilot study. World Neurosurg.

[17] Iorio-Morin C, Ahmed SU, Bigder M (2018). Demographics, interests, and quality of life of Canadian neurosurgery residents. Can J Neurol Sci.

